# Supplementary material for: Transcriptome- and proteome-wide association studies nominate determinants of kidney function and damage
Source: Genome Biol. 2023 Jun 26;24:150. doi: 10.1186/s13059-023-02993-y (PMC10291807; doi:10.1186/s13059-023-02993-y)

Additional file 2: SUPPLEMENTARY FIGURES
for

Integrated Transcriptome- and Proteome-wide Association Studies reveals determinants of kidney function and damage.

Pascal Schlosser[1,*], Jingning Zhang[2], Hongbo Liu[3], Aditya L. Surapaneni[4], Eugene Rhee[5], Dan E. Arking[6], Bing Yu[7], Eric Boerwinkle[7,8], Paul Welling[9,10], Nilanjan Chatterjee[2], Katalin Susztak[3], Josef Coresh[1], Morgan E. Grams[1,11]

*Correspondence: pschlos3@jhu.edu

Table of Contents

FIGURE S1: TWAS QUANTILE-QUANTILE-PLOTS FOR EGFRCR (A), EGFRCYS (B), BUN (C) AND ACR (D) 3

FIGURE S2: TWAS MANHATTAN PLOTS FOR BUN 4

FIGURE S3: TWAS MANHATTAN PLOTS FOR ACR 5

FIGURE S4: PWAS QUANTILE-QUANTILE-PLOTS FOR EGFRCR (A), EGFRCYS (B), BUN (C) AND ACR (D) 6

FIGURE S5: PWAS MANHATTAN PLOTS FOR BUN 7

FIGURE S6: PWAS MANHATTAN PLOTS FOR ACR 8

FIGURE S7: TWAS NOMINATES A CAUSAL IMPACT OF *MUC1* LEVELS FOR KIDNEY FUNCTION AND DAMAGE OBSERVED IN THE GENERAL POPULATION 9

Figure S1: TWAS quantile-quantile-plots for eGFRcr (a), eGFRcys (b), BUN (c) and ACR (d)

Quantile-quantile plots of TWAS p-values were obtained from two-sided z-tests. The diagonal lines and 95% confidence bands are plotted representing the null hypothesis of no association and standard errors under a normal approximation.

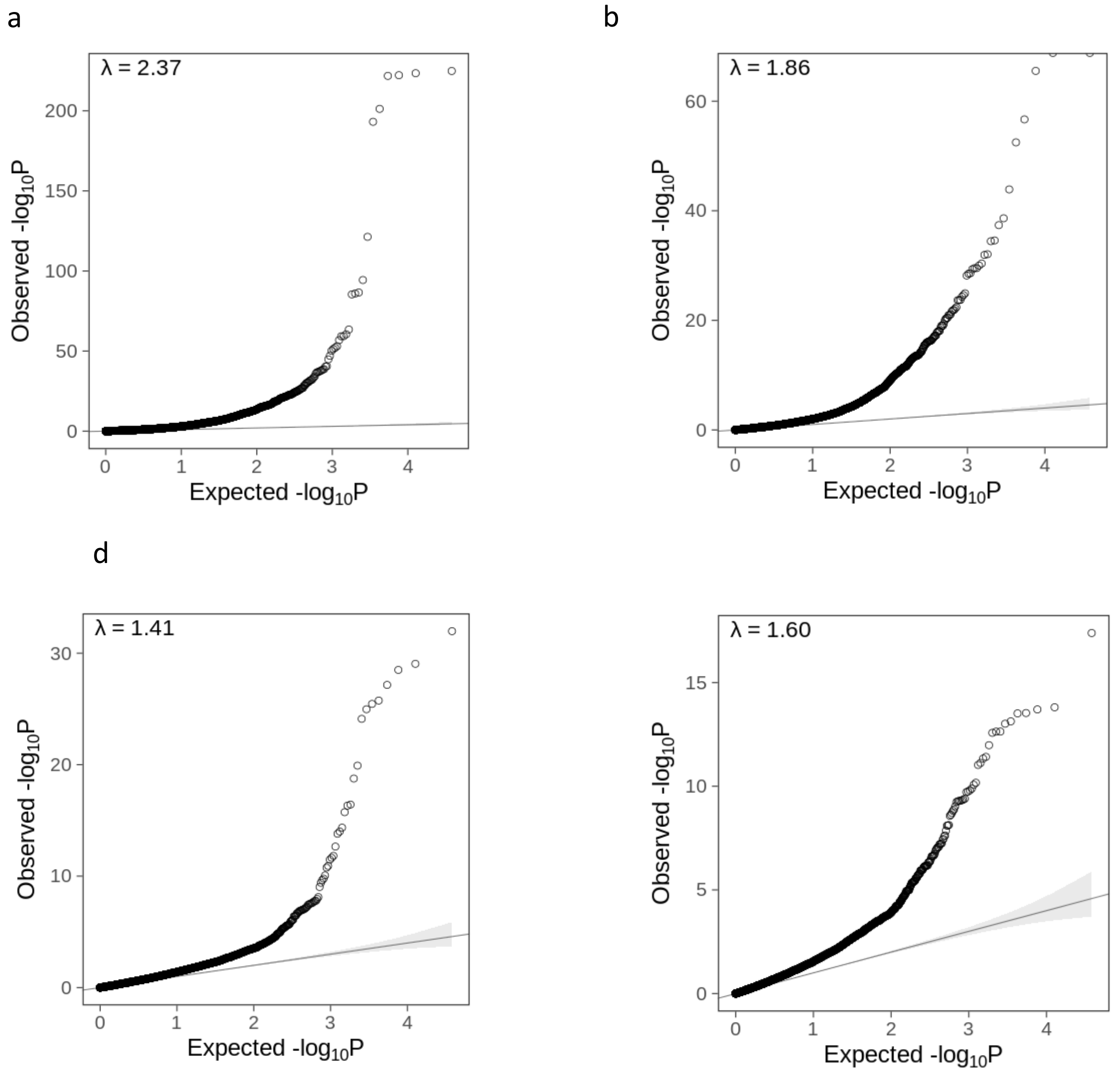

Figure S2: TWAS Manhattan Plots for BUN

Genes that were significant and additionally supported by colocalization analyses of BUN and expression Quantitative Trait Loci (posterior probability >0.8) were labeled. Color code indicates the tissue of the TWAS model. The red lines indicate the Bonferroni adjusted significance threshold (P<3.9e-6).

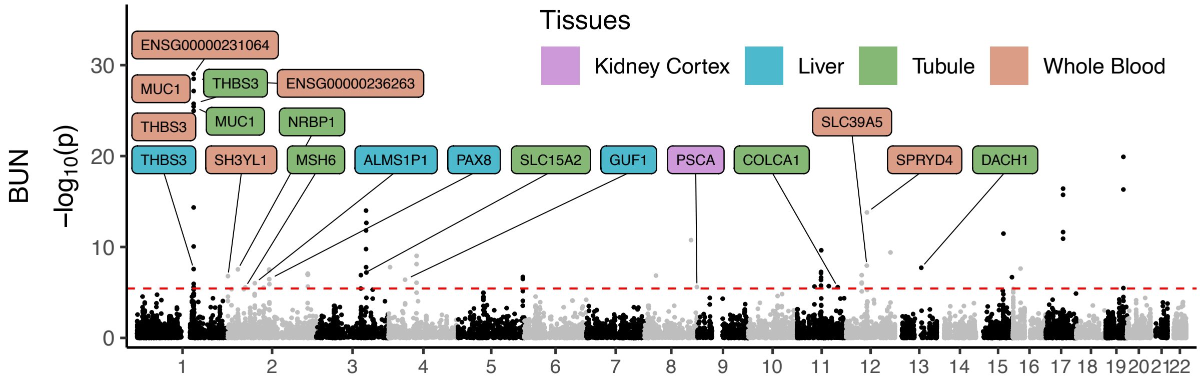

Figure S3: TWAS Manhattan Plots for ACR

Genes that were significant and additionally supported by colocalization analyses of ACR and expression Quantitative Trait Loci (posterior probability >0.8) were labeled. Color code indicates the tissue of the TWAS model. The red lines indicate the Bonferroni adjusted significance threshold (P<3.9e-6).

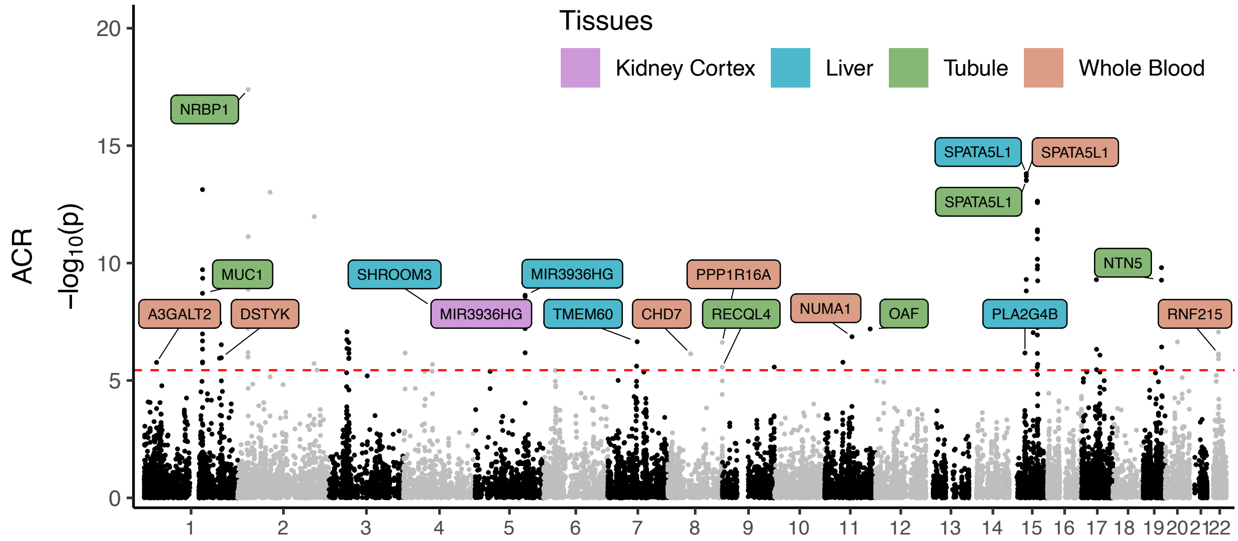

Figure S4: PWAS quantile-quantile-plots for eGFRcr (a), eGFRcys (b), BUN (c) and ACR (d)

Quantile-quantile plots of PWAS p-values were obtained from two-sided z-tests. The diagonal lines and 95% confidence bands are plotted representing the null hypothesis of no association and standard errors under a normal approximation.

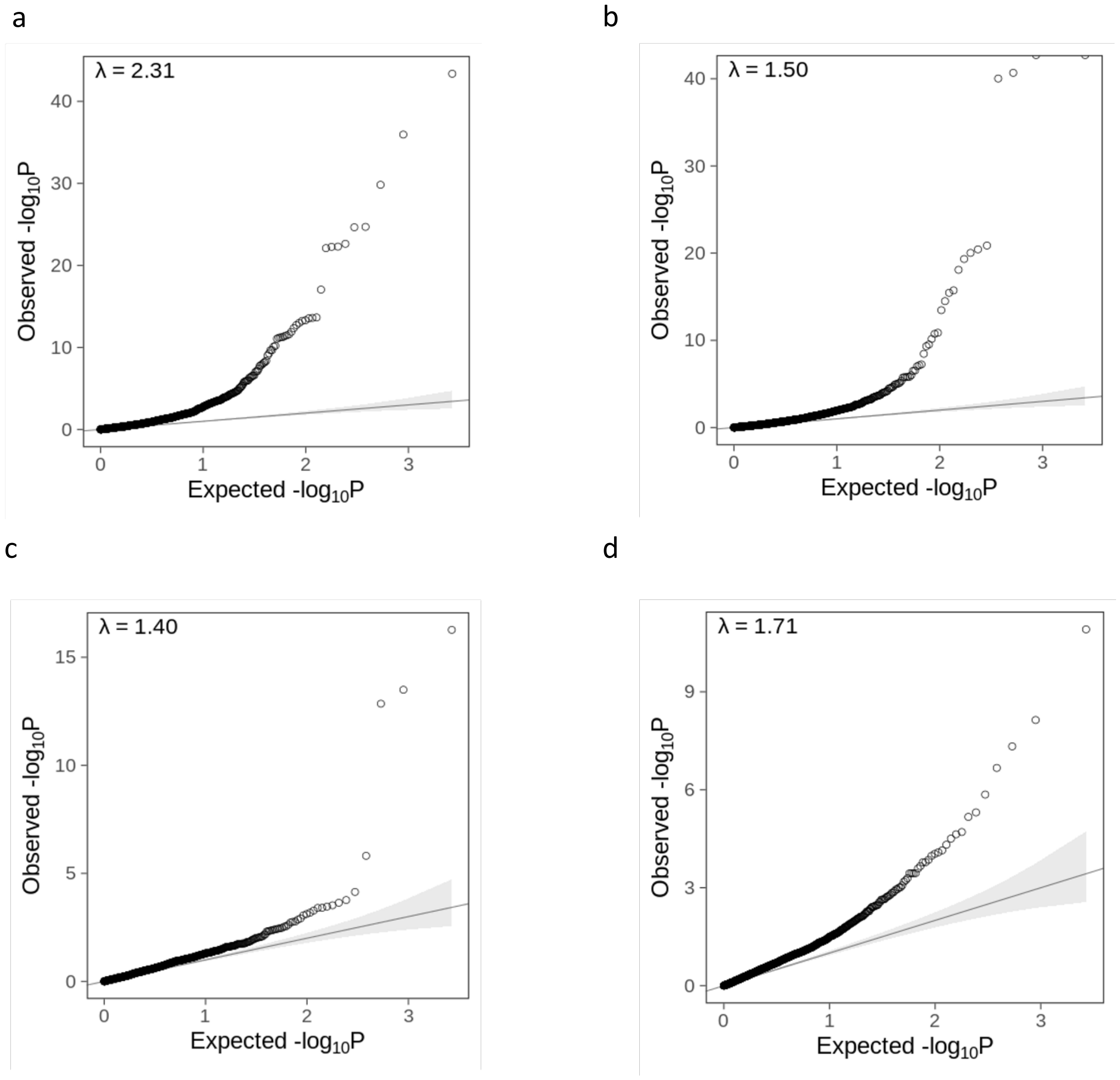

Figure S5: PWAS Manhattan Plots for BUN

The red lines indicate the Bonferroni adjusted significance threshold (P<3.7e-5). Significant proteins were labeled and associations with additional support through colocalization analyses of BUN and protein Quantitative Trait Loci (posterior probability>0.8, Methods) were highlighted in orange.

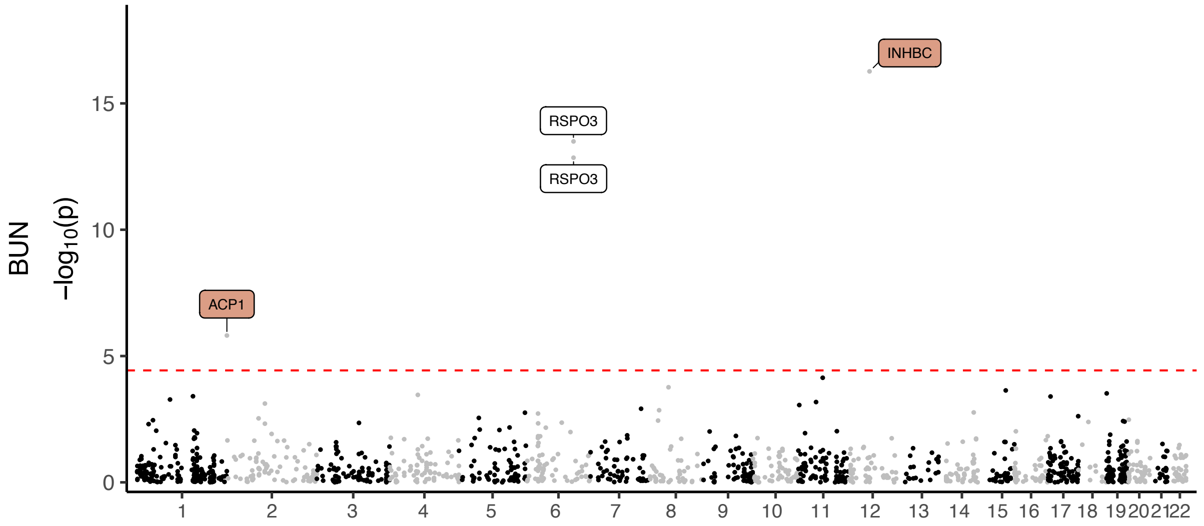

Figure S6: PWAS Manhattan Plots for ACR

The red lines indicate the Bonferroni adjusted significance threshold (P<3.7e-5). Significant proteins were labeled and associations with additional support through colocalization analyses of ACR and protein Quantitative Trait Loci (posterior probability>0.8, Methods) were highlighted in orange.

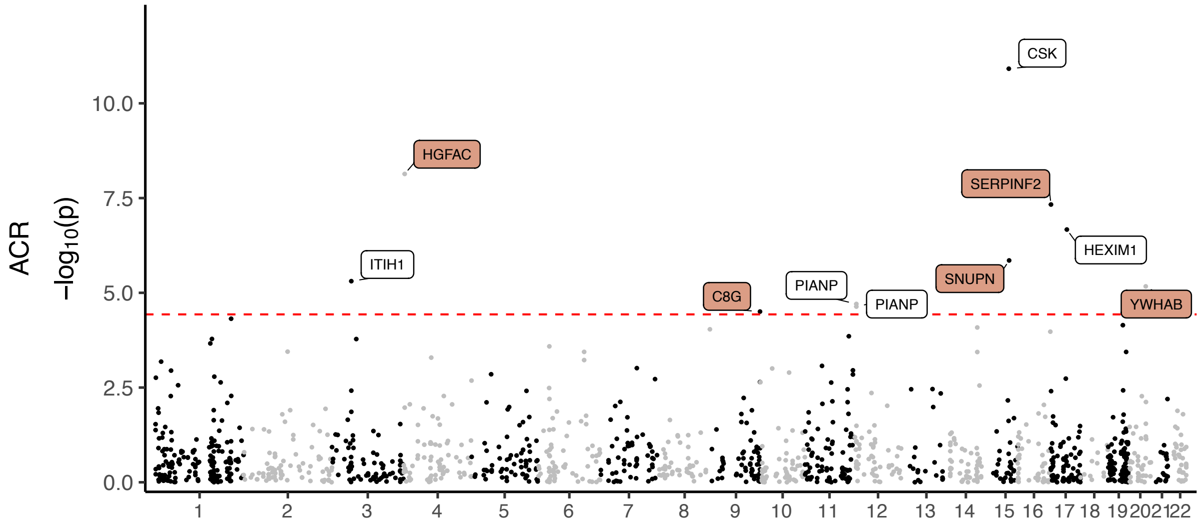

Figure S7: TWAS nominates a causal impact of *MUC1* levels for kidney function and damage observed in the general population

Whole blood and tubule expression models of *MUC1* were significantly associated with all four kidney function and damage traits and supported by colocalization. TWAS models and kidney trait GWAS are based on variation observed in the general population. The two common variant prediction models were moderately related (*cis*-regulated genetic correlation=0.66) and consistently implicated BUN with the strongest association signal. TWAS models are schematically illustrated with the partially overlapping set of SNPs in the *MUC1 cis*-region (1Mb; lines connecting the *MUC1* transcript with tissue specific expression) and different weights (line thickness). The number of variable number of tandem repeats (VNTR) in the second exon is known to be associated with gout and a frameshift in the VNTR with a rare Mendelian kidney disease - autosomal dominant tubule-interstitial kidney disease (ADTKD).

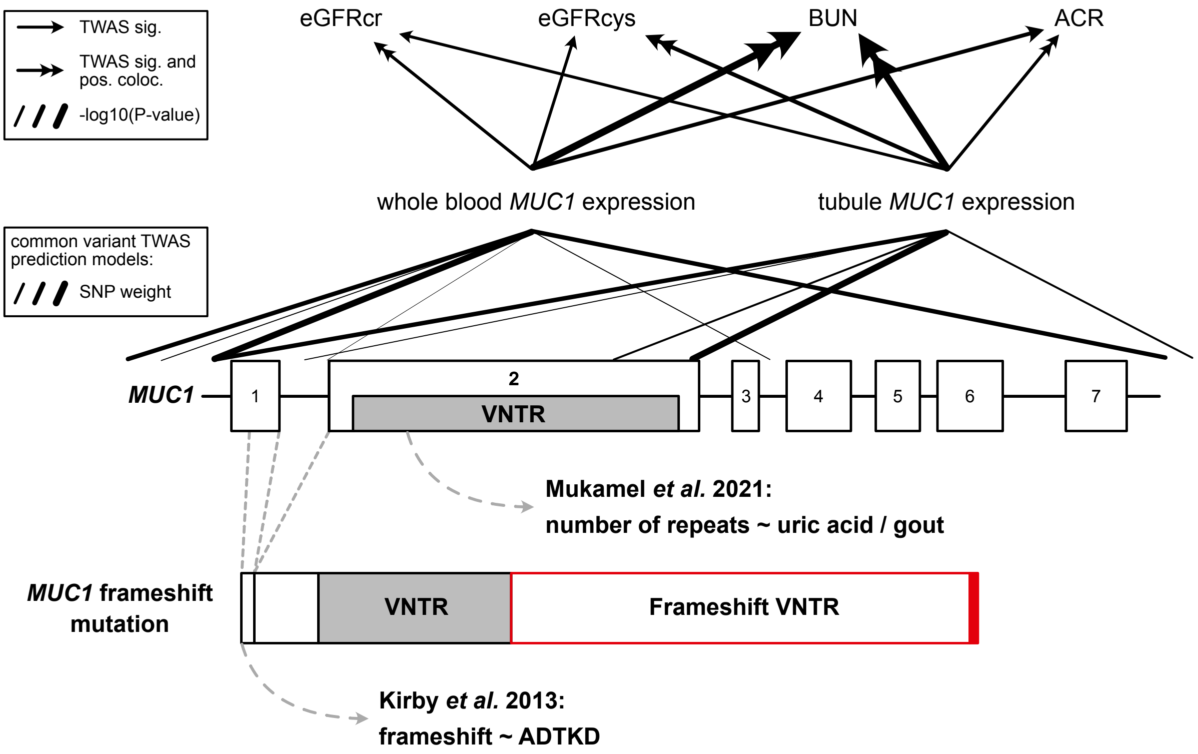

Supplement: Supplementary file 2 — Additional file 2. Supplementary Figures including the QQ-plots, Manhattan plots and the MUC1 TWAS association. [file 13059_2023_2993_MOESM2_ESM.pdf]
